# Molecular characterisation of genital human papillomavirus among women in Southwestern, Nigeria

**DOI:** 10.1371/journal.pone.0224748

**Published:** 2019-11-04

**Authors:** Yewande T. Nejo, David O. Olaleye, Georgina N. Odaibo

**Affiliations:** 1 Department of Biological Sciences, Bowen University, Iwo, Osun State, Nigeria; 2 Department of Virology, College of Medicine, University of Ibadan, Ibadan, Nigeria; University of Cincinnati College of Medicine, UNITED STATES

## Abstract

**Background:**

Persistent infections with high-risk genital Human papillomavirus (HPV) especially types 16 and 18, are associated with cervical cancer. However, distribution of HPV types varies greatly across geographical regions and the available vaccines target only few types. This study was designed to determine the HPV types circulating in Southwestern Nigeria, thereby providing necessary information for effective control of the virus.

**Methods:**

Endocervical swab samples were collected from a total of 295 consenting women attending routine cervical cancer screening, STI clinics and community-based outreach programme. Viral DNA was extracted from the samples and the consensus region of the HPV DNA was amplified by PCR using GP-E6/E7 primers. Type-specific nested multiplex PCR and Sanger sequencing were used to genotype the HPV isolates.

**Results:**

In this study, 51 (17.3%) individuals were positive for HPV DNA using consensus primers that target the E6/E7 genes but only 48 (16.3%) were genotyped. A total of 15 HPV types (HPV-6, 16, 18, 31, 33, 35, 42, 43, 44, 52, 58, 66, 74, 81, 86) were detected, with HPV-31 being the most predominant (32.8%), followed by HPV-35 (17.2%) and HPV-16 (15.5%). Two rare HPV types; 74 and 86 were also detected. The HPV-74 isolate had three nucleotide (CCT) insertions at E7 gene that translated into amino acid proline. Highest nucleotide substitutions (n = 32) were found in HPV-44 genotype. Among positive individuals, 20.8% had dual infections and 86.2% had High-risk HPV types.

**Conclusions:**

Multiple Human papillomavirus types co-circulated in the study. Most of the circulating Human papillomavirus are high-risk type with type 31 being the most predominant. Although the implication of HPV-74 with proline insertion detected for the first time is unknown, it may have effect on the transformation potential of the virus. Polyvalent HPV vaccine will be more effective for the infection control in Nigeria.

## Introduction

Genital Human papillomavirus (HPV) infection is the most frequent sexually transmitted infection globally and most sexually active individuals will be infected with HPV at some periods in their lives [1, 2]. Young women are more vulnerable to the virus and often become infected by multiple HPV types most especially the high-risk types. Infection by these types of genital HPV is recognised as a causal and essential factor for cervical cancer [3]. Human papillomavirus infection is a major health challenge in developing countries where 80% of cervical cancer occurs. In Nigeria, cervical cancer is the second most common cancer and the 2nd cause of female cancer deaths with estimated 14,550 diagnosed cases and 9659 death annually [4].

Human papillomaviruses are small, non-enveloped, epitheliotropic viruses with approximately 8 Kbp circular double-stranded DNA, and belongs to the *Papillomaviridae* family [5]. The genome encodes 6 early proteins (E1, E2, E4, E5, E6 and E7) that are responsible for virus replication, and 2 late proteins (L1 and L2) which are the major and minor viral capsid proteins respectively [6]. About 200 HPV genotypes have been identified based on the sequence of their L1 genes [2, 7]. They can be categorized into cutaneous or mucosal types based upon their tissue tropism [8]. The E6 and E7 proteins are the major oncoproteins which are involved in the transformation and immortalisation of host cells. The E6 and E7 proteins bind and inactivate the two major tumour suppressor proteins; p53 and pRb respectively [9]. The sequences of HPV E6 and E7 regions have been used in the classification of some HPV variants [10–12].

Approximately 40 HPV types infect the female genital tract and are further classified as high-risk HPV (HR-HPV) and low-risk HPV (LR-HPV) types according to their oncogenic potential [13]. Persistent infection with high-risk HPV especially types 16 and 18 are associated with 70% of cervical cancers [14]. Two HPV vaccines (Cervarix and Gardasil) are licensed for use in Nigeria [15] and these target two (HPV-16 and 18) and four (HPV-6, 11, 16 and 18) HPV types respectively [16]. The Nigerian National Cervical Cancer Control Policy in 2010 authorized the vaccination of girls aged 9–15 years with Cervarix, but only few privileged individual have been able to use it due to the low knowledge of HPV infection and vaccines, and high cost of the vaccination [12]. The third HPV vaccine (Gardasil-9) is a nonavalent vaccine that was licensed by FDA in December 2014. It protects against HPV-6, 11, 16, 18, 31, 33, 45, 52, and 58 but it is yet to be licensed in Nigeria [16].

However, there are variations in the distribution of HPV types circulating among Nigerian women in different part of the country which have raised concerns about the effectiveness of the available vaccines in the region. Thus, this study was designed to determine the circulating HPV types among women in Southwestern Nigeria, thereby providing information towards effective prevention and control of HPV infection.

## Materials and methods

### Sample collection and processing

The sample size for this study was determined by using a statistical formula [17], and the expected prevalence used for the calculation was based on the prevalence reported by Thomas *et al*. [18]. Endocervical samples were collected from a total of 295 sexually active women, between ages 23 and 77 years. These include women presenting for routine cervical cancer screening (Pap smear), sexually transmitted infections (STIs) clinic attendees and women enrolled during community based outreach programmes. The participants were enrolled between March, 2014 and November, 2015 from Molete community in Ibadan and two health facilities [University College Hospital (UCH), Ibadan and Baptist Medical Centre (BMC), Saki] all located in Oyo State, Southwestern Nigeria. Women with or without cytological abnormalities or symptoms of STIs were included. On the other hand, women who have undergone hysterectomy, pregnant, or menstruating at the time of sample collection were excluded. Only women who gave informed consent were enrolled for the study.

Socio-demographic, clinical and sexual history were also obtained from each participant using structured questionnaire. Two swab samples were collected from the endocervix of each female participant by inserting Cusco’s speculum into the vagina in order to expose the cervix. Excess mucus was removed from the cervix and surrounding mucosa using cleaning swab. The collection swab was inserted into the endocervix and turned clockwise for 10–15 seconds to ensure adequate sampling. The swabs were removed gently and placed in pre-labelled screw-capped tubes containing 0.5mL of viral transport medium. The samples were carried on ice to the laboratory in the Department of Virology, University College Hospital, Ibadan where they were stored at -80°C until analysed. This study was approved by the University of Ibadan/University College Hospital Institutional Review Committee (UI/UCH IRC) with research approval number UI/EC/12/0387. The result of this study was analysed using IBM SPSS statistic version 21 software. Chi square statistics was used to estimate the degree of correlation between variables with *p* values of <0.05 considered as statistically significant.

### DNA extraction and PCR

**Fig 1:**
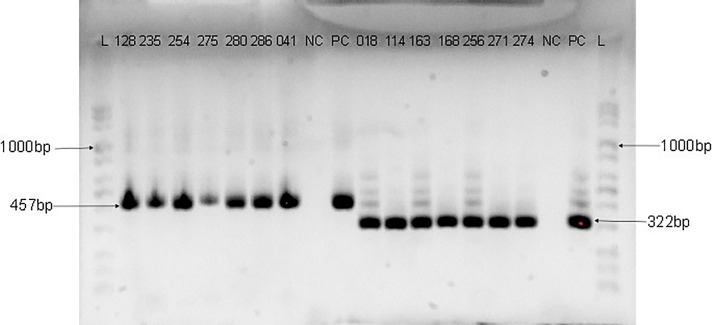
Agarose gel electrophoresis Image of HPV DNA genotyping with HPV-16 (457bp) and 18 (322bp) primers. Numbers are samples ID; L is a mid-range ladder (Jena Bioscience); NC is the negative control while PC is the positive control. The sizes of the PCR products generated are 457bp for HPV-16 and 322bp for HPV-18.

**Fig 2:**
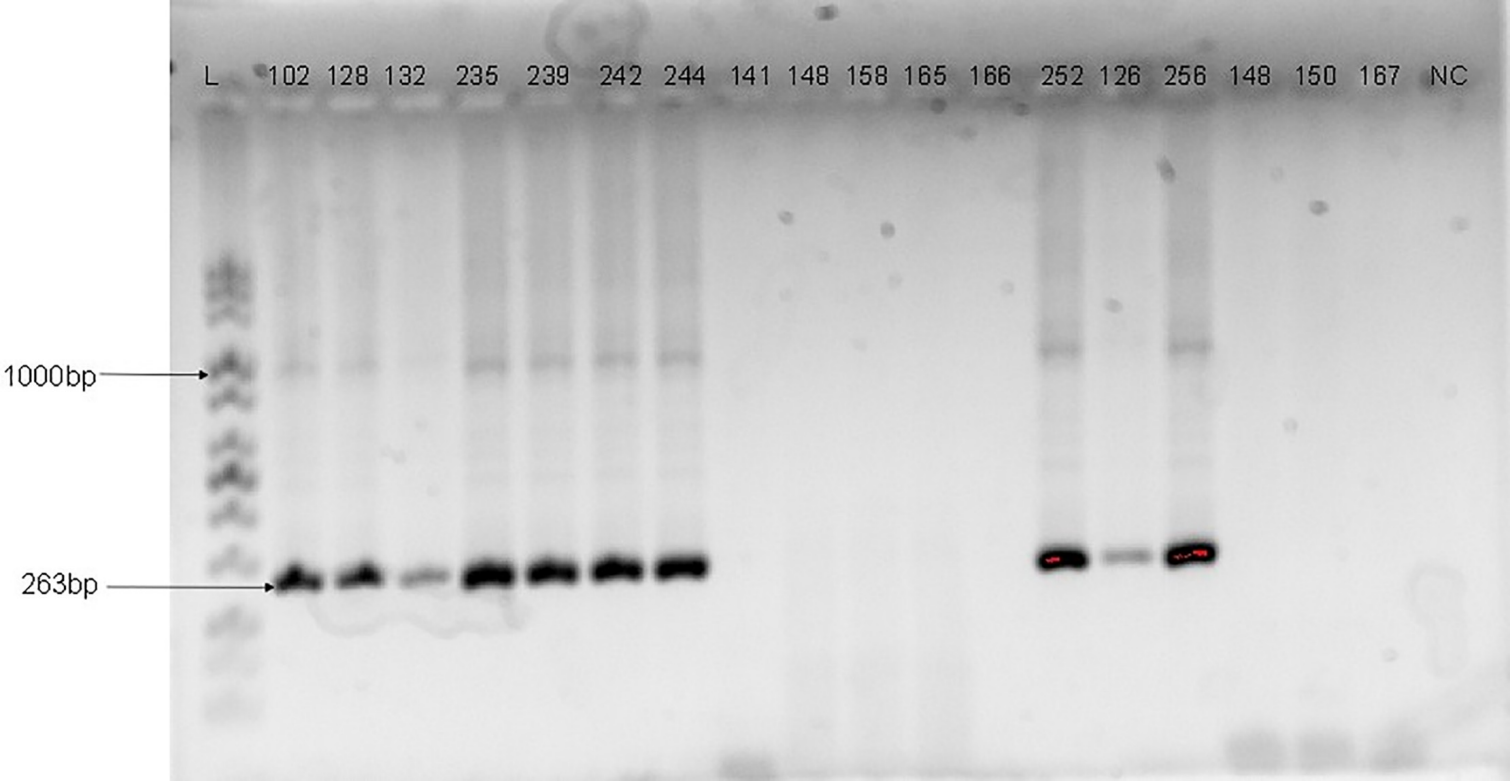
Agarose gel electrophoresis Image of HPV DNA genotyping with HPV-31 E6/E7 primers. Numbers are samples ID; L is a mid-range ladder while NC is the negative control. The size of the PCR products generated for HPV-31 is 263bp.

**Fig 3:**
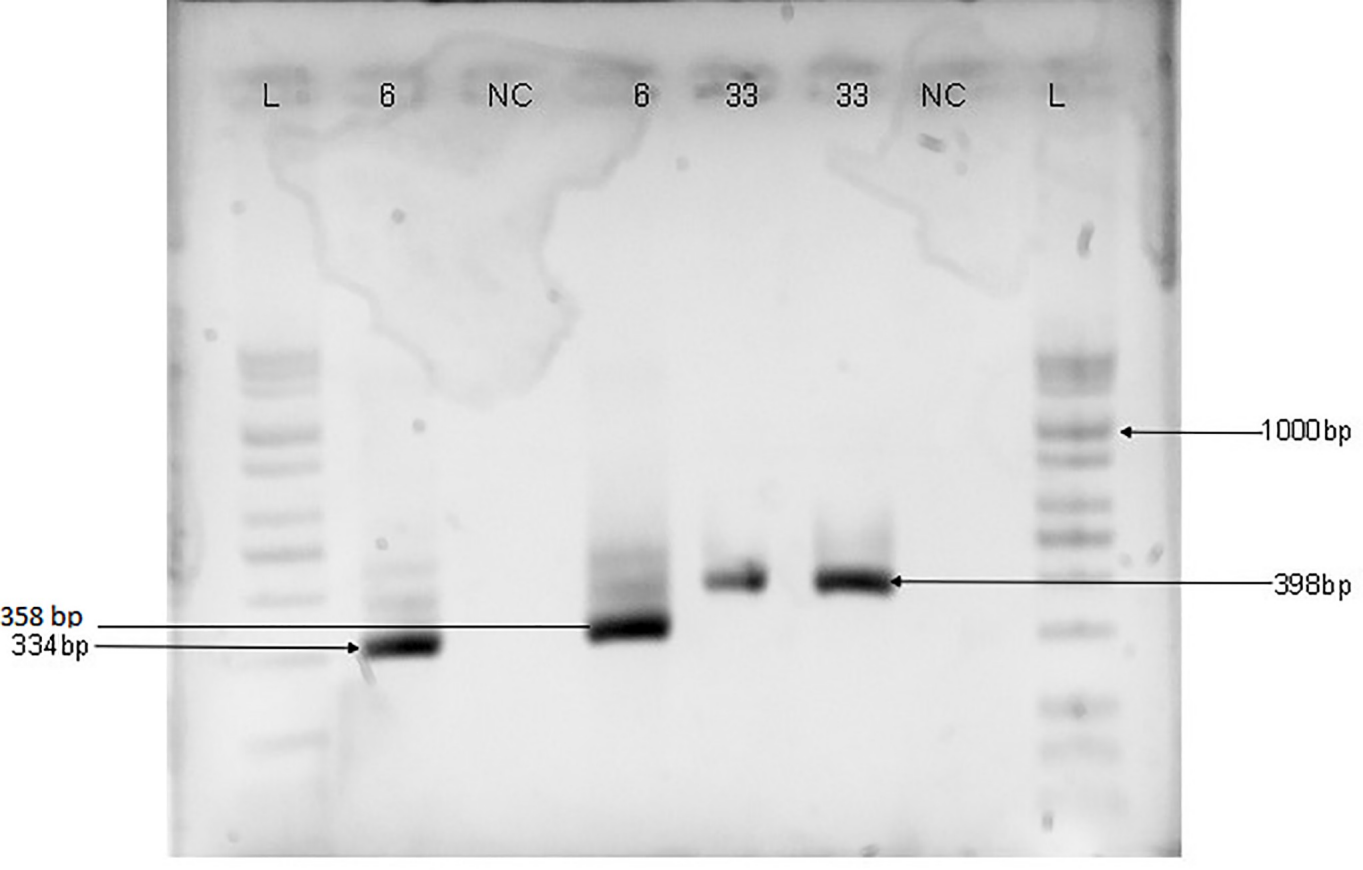
Agarose gel electrophoresis Image of HPV DNA genotyping with HPV-6 (334bp), 35 (358bp) and 33 (398bp) primers. Numbers are samples positive to HPV 6, 35 and 33 as labelled; L is a mid-range ladder while NC is the negative control. The sizes of the PCR products generated are 334bp for HPV-6, 358bp for HPV-35 and 398bp for HPV-33.

Genomic DNA was extracted from each of the samples using commercially available DNA extraction kit (Jena Bioscience, Jena, Germany) according to the manufacturer’s instructions. The consensus region of the HPV DNA was amplified by PCR using primers targeting the E6/E7 gene region [one forward primer (GP-E6-3F) and two back primers (GP-E7-5B and GP-E7-6B)] as previously described by Sotlar *et al*. [19]. The cycling conditions for PCRs with GP-E6/E7 consensus primers were preceded by an initial denaturation step at 95°C for 2 min, followed by 40 amplification cycles of 95°C for 30 s, 47°C for 1 min, and 65°C for 2 min. The last cycle was followed by a final elongation step at 72°C for 10 min. Type-specific primer pairs for HPV-16, 18, 31, 33, 35 and 6/11, were used in a nested multiplex PCR (NMPCR) in two cocktails (16,18,35 and 31,33,6/11) to genotype the HPV isolates [19]. The NMPCRs were performed under the following conditions: the first cycle was preceded by a 2 min denaturation step at 95°C, followed by 35 cycles of 95°C for 30 s, 56°C for 1 min, and 72°C for 1 min and the last cycle was followed by a final elongation step at 72°C for 10 min. All PCRs were performed in a final volume of 25μL reaction mix containing 5μL of the extracted DNA, 5μL of a premix of PCR buffer, dNTPs, Magnesium chloride and Taq Polymerase enzyme in optimized concentration (Jena Bioscience, Germany), and 10 pmol of each primer. Five microliters of the PCR product was used as template for the nested PCRs. The amplified HPV DNA was detected by electrophoresis on 2% agarose gel and visualised using Bio-Rad Gel Doc^™^ XR+System. The size of the PCR products that were generated with GP-E6/E7 consensus primers was 630bp while the length of products amplified with type-specific primer pairs ranged from 263bp to 457bp as shown in Figs 1–3.

### DNA sequencing and phylogenetic analysis

Samples that were not typeable by PCR were sequenced and further analysed for genotype identification. The PCR products were purified with a commercially available PCR purification kit (Jena Bioscience, Germany) according to the manufacturer’s instructions. The sequencing was performed commercially (INQABA BIOTEC, South Africa) on ABI Prism 3130 genetic analyzer (Applied Bio Systems) using the same consensus primers used for the PCR. However, only one of the reverse primers (either GP-E7-5B or GP-E7-6B) was used together with the forward primer (GP-E6-3F) to sequence the DNA samples.

Reference sequences of 179 HPV types available on the International Human Papillomavirus Reference Centre site (https://www.hpvcenter.se/human_reference_clones/) were downloaded and aligned with sequences of the sequenced isolates for phylogenetic analysis. Multiple sequence alignment of the isolates and the reference sequences was carried out using the MUSCLE algorithm on MEGA 6.0.6 software [20]. Evolutionary analyses to estimate divergence between sequences were also conducted on MEGA 6.0.6. The Phylogenetic analysis was inferred using Maximum Likelihood method to generate trees [21]. Multiple alignment and phylogenetic analysis of the isolates’ sequences with existing HPV sequences from other parts of the world were also performed. Nucleotide sequences of 19 isolates were deposited in Genbank and assigned the accession numbers KX545348-KX545366.

## Results

### HPV detection and genotyping

The socio-demographic characteristics of the study participants have been previously described [22]. Out of the 295 samples analysed, 51 (17.3%) were positive for HPV DNA using consensus primers that target the E6/E7 genes but only 48 (16.3%) were genotyped either by type-specific PCR or by sequencing. Human papillomavirus DNAs in 37 samples were genotyped by type-specific primer pairs and identified as HPV-16, 18, 31, 33 and 35. The agarose gel electrophoresis images of the HPV type-specific amplification are shown in Figs 1–3.

Overall, 15 HPV types were detected at various frequencies in 58 infections (Table 1). Some individuals (20.8%) were infected with two HPV types (dual infections) although there were significantly more single infections (79.2%, *p* = 0.001). The six most commonly detected types were HPV-31, 16, 35, 18, 33 and 86. The HPV isolates included 8 high risk types (HPV-16, 18, 31, 33, 35, 52, 58, 66) and 5 low risk types (HPV-6, 42, 43, 44, 81). However, two rare HPV types (HPV-74 and 86) with unclassified risk were also identified. High-risk HPV types were detected more frequently (86.2%) among HPV positive individuals than low-risk types (8.6%).

**Table 1:** Distribution of identified HPV genotypes and types of infection among individuals tested.

HPV Risk Type	No. positive (%)	HPV Infection type	No. positive (%)
**High-Risk (HR)**		**Single**	
HPV 16	9 (15.5)	HPV 16	6 (12.5)
HPV 18	7 (12.1)	HPV 18	3 (6.3)
HPV 31	19 (32.8)	HPV 31	13 (27.1)
HPV 33	2 (3.4)	HPV 33	2 (4.2)
HPV 35	10 (17.2)	HPV 35	6 (12.5)
HPV 52	1 (1.7)	HPV 52	1 (2.1)
HPV 58	1 (1.7)	HPV 58	1 (2.1)
HPV 66	1 (1.7)	HPV 66	1 (2.1)
Subtotal	50 (86.2)	HPV 6	1 (2.1)
**Low-Risk (LR)**		HPV 43	1 (2.1)
HPV 6	1 (1.7)	HPV 44	1 (2.1)
HPV 42	1 (1.7)	HPV 86	2 (4.2)
HPV 43	1 (1.7)	Subtotal	38 (79.2)
HPV 44	1 (1.7)		
HPV 81	1 (1.7)	**Dual**	
Subtotal	5 (8.6)	16 & 31	2 (4.2)
**Unclassified Risk (UR)**		16 & 35	1 (2.1)
HPV 74	1 (1.7)	18 & 31	2 (4.2)
HPV 86	2 (3.4)	18 & 42	1 (2.1)
Subtotal	3 (5.2)	18 & 74	1 (2.1)
**Total Infections**	**58 (100)**	31 & 35	2 (4.2)
		35 & 81	1 (2.1)
**HR & HR**	7 (2.4)	Subtotal	10 (20.8)
**HR & LR**	2 (0.7)		
**HR & UR**	1 (0.3)	**HPV Positive**	**48 (16.3)**
**HR only**	33 (11.2)	**HPV Negative**	**247 (83.7)**
**LR only**	3 (1.0)	**Untypeable**	**3 (1.0)**
**UR only**	2 (0.7)	**Total**	295 (100)

Nine HPV types (HPV-31, 35, 16, 18, 33, 58, 74, 86 and 66) were detected among 26 apparently healthy individuals. However, 11 HPV types (31, 35, 16, 18, 52, 6, 42, 43, 44 and 81) were detected among 22 participants with clinical symptoms of STI (Table 2). There was no significant difference in the HPV types that infected the two groups (*p =* 0.518). High-risk HPV (types 31, 35, 16 and 18) were found among both groups of participants. All the detected low risk types were found among symptomatic individuals (*p =* 0.007).

**Table 2:** Distribution of HPV types by health status of individuals tested.

Apparently Healthy Individuals (n = 26)			Individuals with symptoms of STI (n = 22)		
HPV Type	No. of Infection	% of Infection	HPV Type^a^	No. of Infection	% of Infection
HPV-31	10	32.3	HPV-31	9	33.3
HPV-35	8	25.8	HPV-16	4	14.8
HPV-16	5	16.1	HPV-18	5	18.5
HPV-18	2	6.5	HPV-35	2	7.4
HPV-33	2	6.5	HPV-52	1	3.7
HPV-58	1	3.2	HPV-6	1	3.7
HPV-66	1	3.2	HPV-42	1	3.7
HPV-74	1	3.2	HPV-43	1	3.7
HPV-86	1	3.2	HPV-44	1	3.7
			HPV-81	1	3.7
			HPV-86	1	3.7
**Total**^**b**^	**31**	**53.4**		**27**	**46.6**

STI; Sexually transmitted infection

^a^Low-risk HPV types are highlighted

^b^Total number of infections (some individuals were infected with multiple types)

### HPV sequencing analysis

Fourteen HPV types (HPV-6, 16, 18, 31, 35, 42, 43, 44, 52, 58, 66, 74, 81 and 86) were identified by sequence analysis from 19 sequenced samples. Each HPV type has unique nucleotide and amino acid differences (Tables 3 and 4).

**Table 3:** HPV E6 sequences of study isolates with their nucleotide substitutions and amino acid changes.

S/N	Name	HPV Type	E6 Size	^a^E6 Nucleotide Substitutions	E6 Amino acid change
1	**NGIb106-52**	HPV-52	102-548nt (447bp)	T404C; C506G	No mutation
2	**NGIb128-31**	HPV-31	108-557nt (450bp)	**C285T**; A320T; G404A; A407G; **C520T**	H60Y; A138V
3	**NGIb168-42**	HPV-42	114-566nt (453bp)	G284A; T310G; **T311G**	F66W
4	**NGSk203-58**	HPV-58	110-559nt (450bp)	C187T; **G245C**; C307T; T322C; **C367A**; **A398G**	V46L; D86E; N97D
5	**NGSk241-86**	HPV-86	1-447nt (447bp)	**T63A**; **G71A**; T372C; A449C	D21E; S24N
6	**NGSk246-81**	HPV-81	102-566nt (465bp)	**C269G**; **C391T**	N56K; T97I
7	**NGSk256-18**	HPV-18	105-581nt (477bp)	T251C; G266A; C287G; G374A; T485C; **C491A**; A548G; C549A	N129K
8	**NGSk260-35**	HPV-35	110-559nt (450bp)	C131A; A295T	No mutation
9	**NGSk266-6**	HPV-6	102-554nt (453bp)	A221T; **C251G**; A323C; A365T; C392T; G473A; C479T	H50Q
10	**NGSk270-44**	HPV-44	105-557nt (453bp)	**T129G**; A180C; **C183T**; G215C; A218G; **G220C**; T221C; **A268T**; C269T; A275T; **C312G**; **A345G**; **A349T**; **G363T**; A380G; **T386G**; **A387C**; C396T; C405T;G407C^**b**^; C416T; **C470G**; C482T; A494G; **G495A**; T524C; A548C; A556G	S9A; N26H; S39T; Y55F; L70V; N81D; Y82F; V87L; N94K; K95Q; L101F; D122E; D131N
11	**NGSk271-74**	HPV-74	1-453nt (453bp)	**G35C**; A129T; **A148G**; C331T; **T361G**	S12T; N50D; L121V
12	**NGSk274-18**	HPV-18	105-581nt (477bp)	C287G; A476G; T485C; C549A	No mutation
13	**NGSk275-16**	HPV-16	83-559nt (477bp)	T109C; **G132T**; **C143G**; G145T; T286A; A289G; **C335T**; A403G	R17I; Q21D; H85Y
14	**NGSk277-86**	HPV-86	1-447nt (447bp)	**T63A**; **G71A**	D21E; S24N
15	**NGSk278-16**	HPV-16	83-559nt (477bp)	**C88G**; T109C; **G132T**; **C143G**; G145T; T286A; A289G; **C335T**; A403G	H2Q; R17I; Q21D; H85Y
16	**NGSk280-16**	HPV-16	83-559nt (477bp)	T109C; **G132T**; **C143G**; G145T; T286A; A289G; **C335T**; A403G	R17I; Q21D; H85Y
17	**NGSk282-43**	HPV-43	102-569nt (468bp)	**A105T**; T218G; **A256G**; T380A; C513T; **A543C**	T2S; K52R; S148R
18	**NGSk291-16**	HPV-16	83-559nt (477bp)	T109C; **G132T**; **C143G**; G145T; T286A; A289G; **C335T**; A403G	R17I; Q21D; H85Y
19	**NGSk294-66**	HPV-66	102-569nt (468bp)	**T108C**; C234T	S3P

^a^nucleotide substitutions in bolds translated into different amino acid respectively

^b^the two highlighted nucleotide substitutions translated into only one amino acid change

**Table 4:** HPV E7 sequences of study isolates with their nucleotide substitutions and amino acid changes.

S/N	Name	HPV Type	E7 Size	E7 Nucleotide^a^ Substitutions	E7 Amino acid change
1	**NGIb106-52**	HPV-52	553-626nt (74bp)	No substitution	No mutation
2	**NGIb128-31**	HPV-31	560-657nt (98bp)	G580A; C646T	No mutation
3	**NGIb168-42**	HPV-42	542-638nt (97bp)	C607T; A628G; G634A	No mutation
4	**NGSk203-58**	HPV-58	574-657nt (84bp)	No substitution	No mutation
5	**NGSk241-86**	HPV-86	423-478nt (56bp)	**A449C**	K9N
6	**NGSk246-81**	HPV-81	542-628nt (87bp)	C616T; G625A; C626T	No mutation
7	**NGSk256-18**	HPV-18	590-683nt (94bp)	**C593T**	H2Y
8	**NGSk260-35**	HPV-35	562-644nt (83bp)	**G595T**	V12F
9	**NGSk266-6**	HPV-6	530-632nt (103bp)	G617C; T618C; **A619T**	V30P
10	**NGSk270-44**	HPV-44	533-619nt (87bp)	**A548C**; A556G; **C572G**; **A580T**	T6P; Q14E; E16D
11	**NGSk271-74**	HPV-74	429-526nt (98bp) Insertion of CCT	Insertion of CCT^**b**^ at A512**CCT**513G	Insertion of P at L28**P**29D
12	**NGSk274-18**	HPV-18	590-620nt (31bp)	**T612C**	L8S
13	**NGSk275-16**	HPV-16	562-646nt (85bp)	A646G	No mutation
14	**NGSk277-86**	HPV-86	423-507nt (85bp)	No substitution	No mutation
15	**NGSk278-16**	HPV-16	562-648nt (87bp)	A646G**; T648C**	N29D
16	**NGSk280-16**	HPV-16	562-646nt (85bp)	A646G	No mutation
17	**NGSk282-43**	HPV-43	530-614nt (85bp)	**A543C**	K5T
18	**NGSk291-16**	HPV-16	562-646nt (85bp)	A646G	No mutation
19	**NGSk294-66**	HPV-66	572-659nt (88bp)	A586T; G628T; G658A	No mutation

^a^nucleotide substitutions in bolds translated into different amino acid respectively

^b^insertion of CCT (highlighted) translated to Proline (P)

**Fig 4:**
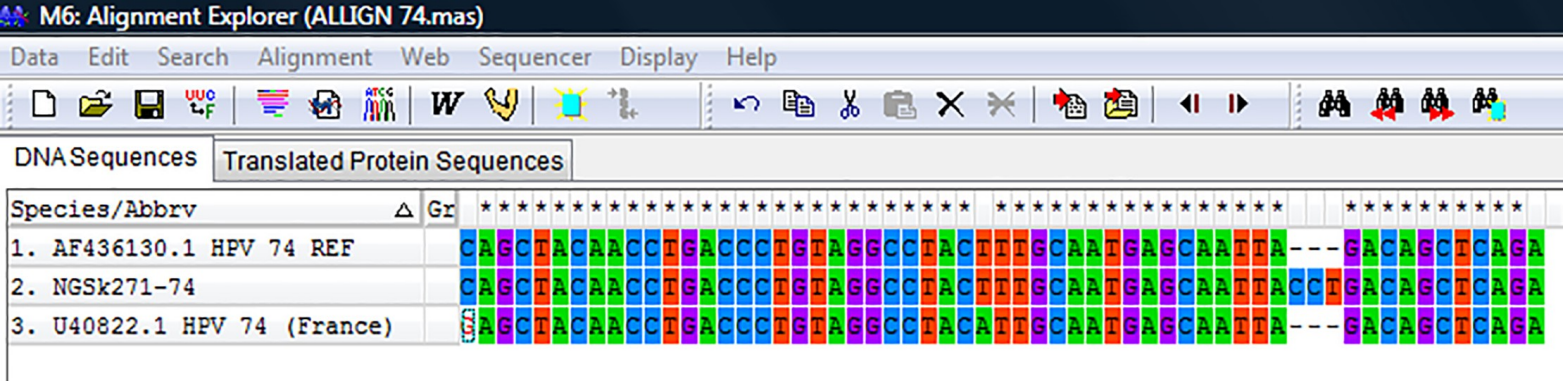
Nucleotide sequence alignment of HPV-74 isolate with reference sequence and sequence from France, showing insertion of CCT. The top row specifies the positions where variations were observed in the sequences; positions without variations were marked with an asterisk (*) while no asterisk indicated a variation position. The letters indicated their nucleotide base sequences.REF; Reference sequence.

**Fig 5:**
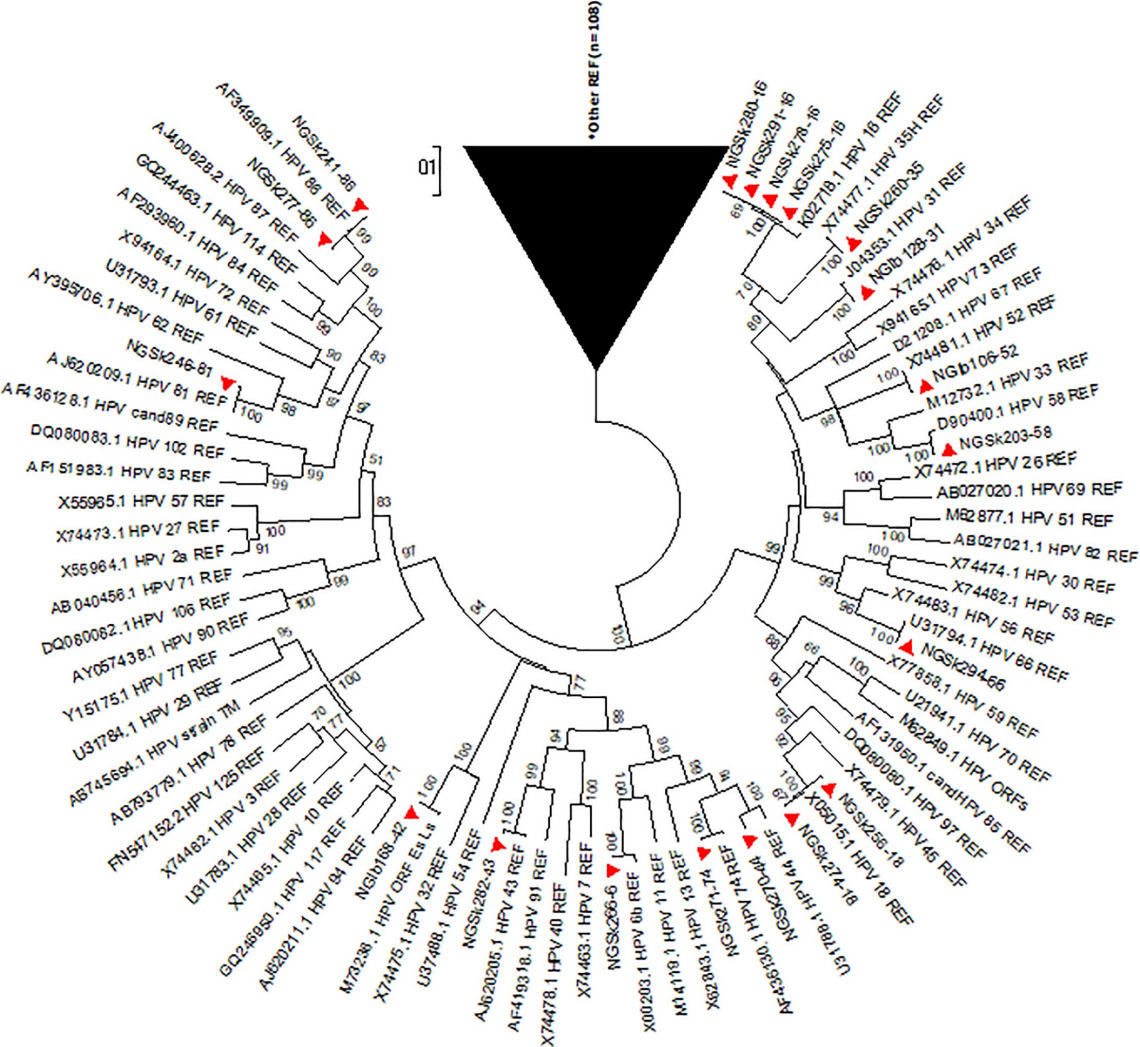
Phylogenetic tree of sequences of study isolates with HPV reference sequences. All the nineteen study isolates clustered with only fourteen HPV reference sequences (REF). Study isolates are indicated by shaded triangles. *REF; Reference Sequences.

**Fig 6:**
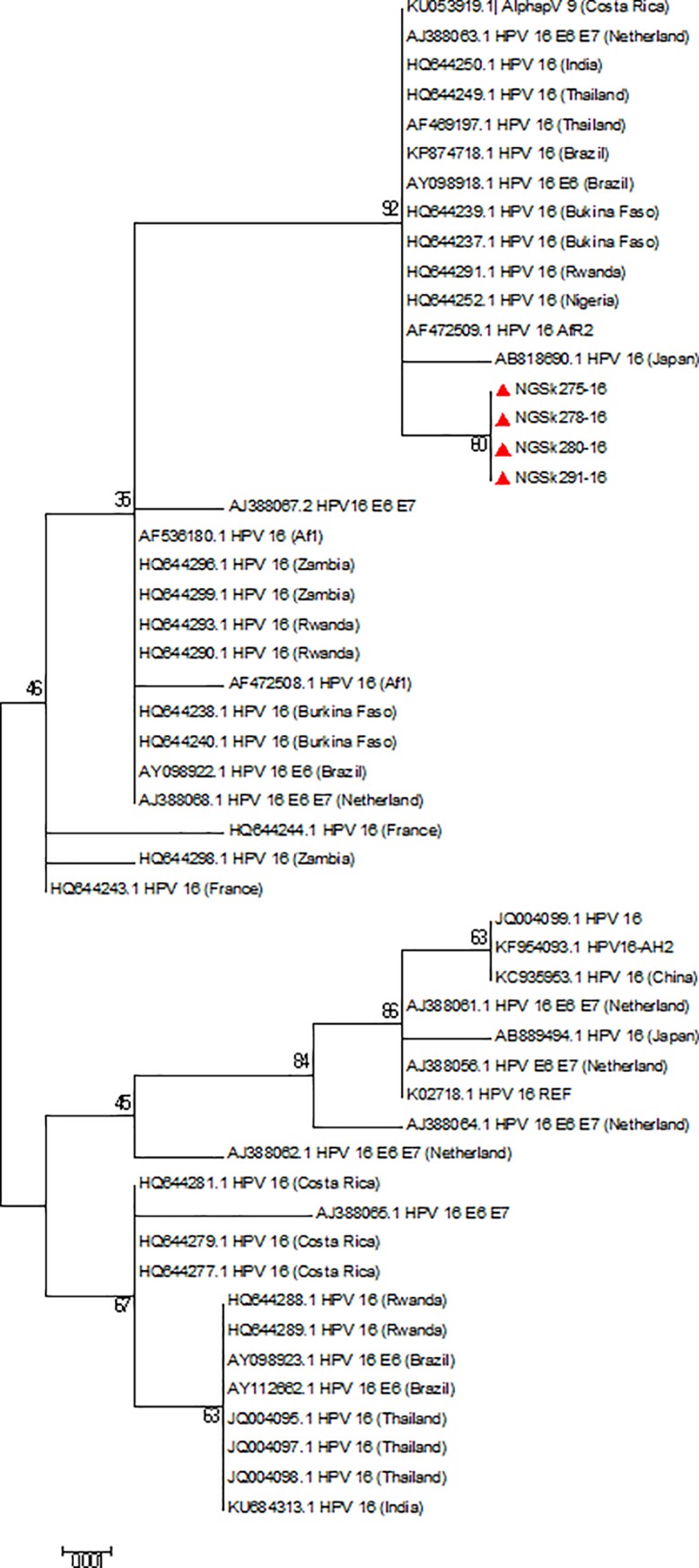
Phylogenetic tree of HPV-16 isolates. All the four HPV-16 study isolates clustered with African 2 variants on the phylogenetic tree. Study isolates are indicated by shaded triangles; AF1: African 1 variants; AFR2: African 2 variants.

**Fig 7:**
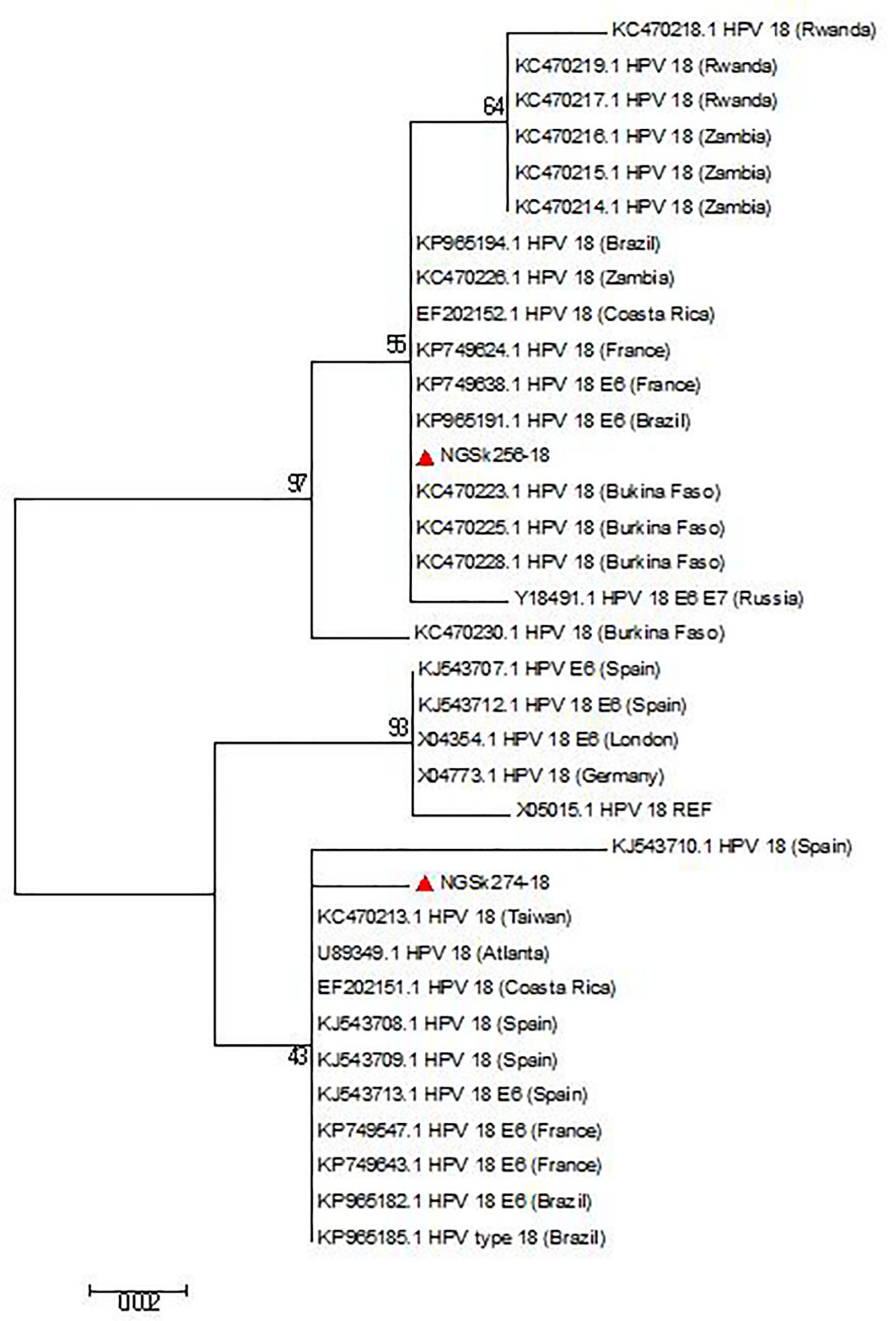
Phylogenetic tree of HPV-18 isolates. The two HPV-18 study isolates clustered differently on the phylogenetic tree. Study isolates are indicated by shaded triangles.

**Fig 8:**
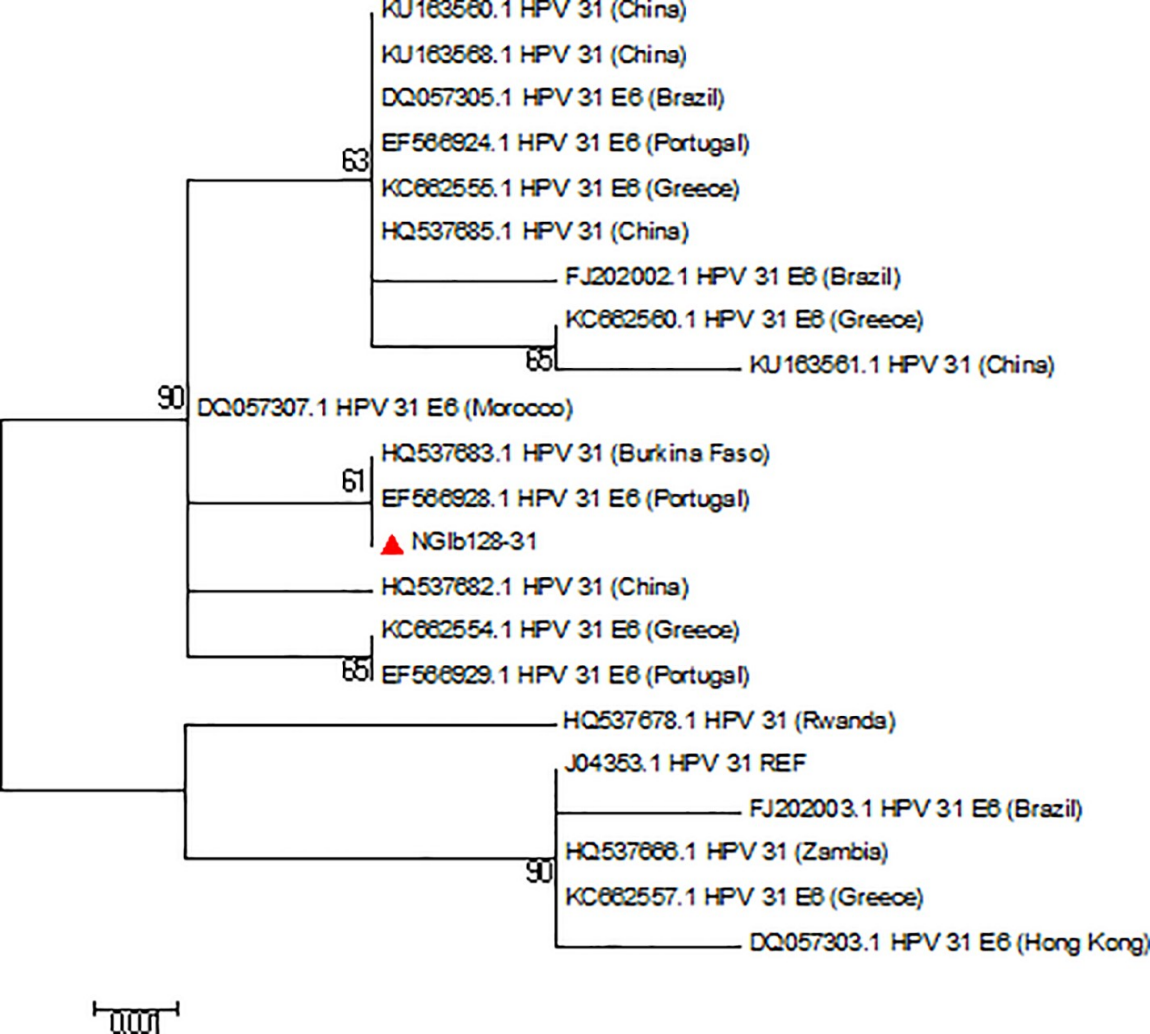
Phylogenetic tree of HPV-31 isolates. The HPV-31 study isolate clustered with isolate from Portugal. The isolate is indicated by a shaded triangle.

The overall mean distance within the isolates was 0.659±0.035. HPV 44 sequence had the highest nucleotide variations both at the E6 and E7 region (n = 32). The HPV-74 sequence had an insertion of a triplet codon **(CCT)** at the E7 region between A512 and G513 position of the reference sequence (Fig 4) that translated to insertion of amino acid Proline (**P)** between L28 and D29 position. The phylogenetic trees of sequences of the study isolates with their reference sequences are shown (Figs 5–8).

## Discussion

This study is a cross-sectional study of Human papillomavirus in Nigeria. Fifteen HPV types (HPV-6, 16, 18, 31, 33, 35, 42, 43, 44, 52, 58, 66, 74, 81 and 86) were detected in this study indicating co-circulation of multiple HPV types in Southwest Nigeria. The prevalence of high-risk HPV (HR-HPV) type was significantly higher than the low-risk type. The high prevalence of high-risk type in this study is a cause for concern because persistent infections with these types have been recognised as a necessary cause for cervical cancer. Similar results of higher prevalence of HR-HPV have been previously reported in several studies in Nigeria [18, 23–29]. Related findings have also been documented in some other African countries [30–33].

There were significantly more single infections (79.2%) in this study than dual infections (20.8%) which corroborates the findings of past studies [34, 30]. Nonetheless, infections with multiple high-risk HPV types may pose greater risk of developing cervical cancer. Multiple HPV infections could affect HPV testing especially when assay used is not able to detect other types present in multiple infections and this could lead to HPV type-specific prevalence being under reported. Moreover, it would be difficult to achieve effective immunisation against HPV infection where the available vaccines are only able to protect against some HPV types leaving others circulating in the population.

The predominant HPV type in this study is HPV-31, followed by HPV-35, 16, 18, 33 and 86. HPV-31 has been reported to be very common in Europe and Latin America [35]. The reason for its predominance in this study is not fully understood. Howbeit, the analysis of the travel history of the participants showed that 63.2% of individuals infected with this HPV type have at one point of their life travelled outside Nigeria, although the exact travel locations were not captured. The only sequenced HPV-31 also clustered with sequence from Portugal. Therefore, it is not impossible that the infection was acquired through their sexual interactions with infected individuals outside of Nigeria. The prevalence of HPV-31 in this study (4.4%) seemed comparable with 4.0% and 3.8% as previously reported [36, 37]. Similar prevalent HPV types were reported among women in a global study but with HPV-16 predominating [34, 35]. Epidemiological studies among sub-Saharan African women also showed that HPV-16, 18, 45, 35, 33 and 52 are the most commonly detected types [38]. However, some African studies revealed variations in the prevalent HPV types that were also different from the types detected in this study [34, 39, 33].

Several studies in Nigeria have shown disparities in the prevalent HPV types. In Lagos, HPV type 31 also predominated albeit among HIV positive individuals, followed by types 52, 53 and 35 while among HIV negative group, type 18, 16, 52 and 56 were the commonest [27]. The most prevalent types found among women in Port Harcourt [40] and Ibadan [18] agrees with the result of this study except for HPV-18 and 16 dominating respectively. Some factors that could be responsible for variations in the distribution of HPV types across the world include: the type of assay used, multiple HPV infections, differences in the study population, and varying exposures of individuals to different risk factors in different geographical regions [41].

High-risk HPVs found both among apparently healthy individuals and symptomatic participants further buttress the fact that most infections with HPV do not manifest clinically. Routine HPV testing among general population would therefore be important for diagnosis. Further studies on the rare types (HPV-74 and 86) detected in this study would be essential to access their distribution and determine their association with cervical carcinoma. All the low-risk HPV types detected were found among symptomatic individuals, although, only one individual out of those with genital wart had low-risk HPV (type 81). This could imply that HPV types other than low-risk types may also be associated with genital warts. Hence, regular HPV testing and not the mere presence of a wart will be required to detect HPV types in an infected individual.

The phylogenetic analysis of the 19 sequenced isolates showed that the E6 and E7 sequences of HPV types are conserved within the types but varies between types. The overall mean distance between the isolates being 0.659 showed a high level of diversity among the isolates in the E6/E7 region. To the best of our knowledge; this seems to be the first study that describes the sequences of HPV types by E6/E7 genes among Nigerian women.

The analysis of HPV E6 and E7 sequences has been used to determine the variants of some HPV types [10–12]. Although the specific effects of HPV variants on cervical carcinogenesis is still poorly understood, several reports suggested that variants of some high-risk HPVs especially types 16 and 18 may be more strongly associated with persistence and progression of lesions to cervical cancer [10–12]. Four of the sequenced isolates were HPV-16 and are identified as variants of African lineage (Af) as a result of 3 missense mutations (R17I, Q21D and H85Y) found in their E6, as previously described [42–45]. The G132T and A403G substitutions also observed in these isolates identified them as Af-2a sub-lineage group as described [10, 45–47] or lineage B [48, 49]. Literatures have shown that African lineages are the most prevalent in African regions [42, 50, 51] and are more highly oncogenic than the European variants [52–54]. The Q21D alteration has been shown to increase the affinity of E6 with p53 by 180% [55] while R17I could lead to decreased affinity with p53 and its degradation [56, 57]. Although the effect of missense mutation (N29D) found in isolate NGSk278-16 was not studied, previous studies [58, 45] have shown that mutation at this site (N29S) alters the affinity of E7 for pRB and modifies its oncogenic potential.

The two HPV-18 sequenced in this study have 3 silent mutations at their E6 regions as previously found [59, 60]. Isolate NGSk256-18 belongs to the HPV-18 Af lineage (now B lineage) based on the presence of A548G in addition to a C549A [46] while isolate NGSk274-18 belongs to A lineage due to C549A substitution. The HPV-6 E6 nucleotide alterations observed in this study were previously found among HPV-6a group [61–63] or HPV-6 sublineage B3 [48]. Substitutions at the E6 region of HPV-31 study isolate have been previously described as mutations that occur in HPV-31 variants lineage C [64–66]. However, the association of these variants with morbidity and progression to cancer is unknown.

The HPV-44 sequence with the highest nucleotide substitutions had only two of the E6 substitutions (C183T and C396T) reported by Maver *et al*. [67]. The isolate clustered mostly with HPV-55 now considered a subtype of HPV-44 [68, 8]. The E6/E7 sequences of other sequenced types (HPV-35, 42, 52, 58, 66 and 81) have been described by fewer studies [65, 69, 70, 66, 59] but were not linked to any variant type.

HPV-86 and 74 sequenced in this study are very rare. Their prevalence has been reported by only limited studies [71–73]. The two HPV-86 (isolates NGSk241-86 and NGSk277-86) clustered differently on the phylogenetic tree. The HPV-74 sequence had a triplet codon **(CCT)** insertion that translated to amino acid Proline (**P)** at the E7 region. Based on information available in the literature, this is the first report of the insertion. Although the implication of the insertion is unknown, it may have effect on the oncogenic potential of the virus.

The presence of HPV-74 and 86 among participants in this study could not be linked to any factor. However, the genome of HPV-74 was first identified from an immunosuppressed woman with persisting low-grade vaginal intraepithelial neoplasia [74], while that of HPV-86 was initially isolated from the cervicovaginal cells of a woman with cervical intraepithelial neoplasia grade 1 [75]. Future studies that will detect HIV infection and cytological abnormalities of all participants will be conducted in addition especially to access the association of these rare types with cervical abnormalities.

In this study, 3 (5.9%) out of the 51 positive samples could not be successfully genotyped. The three samples had unreadable sequences due to mixed peaks shown on their sequenced data which means that it is still possible to have some more HPV types in the samples. Hence, the genotypic result of this study might not be representative of the overall circulating genotypes in Southwest Nigeria. This limitation however can be tackled in future studies by the use of Next Generation Sequencing technology.

In conclusion, the high prevalence of high-risk HPV types detected in this study even in multiple infections reveals the burden of HPV infection in the country. Infections with high-risk types have the tendency of progressing to malignancy; thus an increased surveillance to determine the women at risk of cervical cancer is advocated. Further studies into detecting HPV-74 and 86 (rare types) will be important to determining their association with cancer. Multiple HPV types detected with non-HPV 16 and non-HPV 18 dominating indicated that HPV-16 and 18 might not be the major circulating types in Nigeria and the available vaccines in the country may be less effective in controlling the infection. However, 7 (HPV-6, 16, 18, 31, 33, 52 and 58) out the 15 circulating types, detected in 40 infections (69.0%) are vaccine types in Gardasil-9. It is therefore necessary for policy makers to consider a more protective polyvalent vaccine like Gardasil-9 for effective control of HPV infection, and the vaccine should be made available at a subsidised rate to the target population in Nigeria.

## References

[1] Georgieva S, Iordanov V, Sergieva S. Nature of cervical cancer and other HPV-associated cancers. Journal of BUON. 2009; 14: 391–398. 19810128

[2] Bernard HU, Burk RD, Chen Z, van Doorslaer K, Hausen HZ, de Villiers EM. Classification of papillomaviruses (PVs) based on 189 PV types and proposal of taxonomic amendments. Virology. 2010; 401(1): 70–79. 10.1016/j.virol.2010.02.002 20206957 PMC3400342

[3] Bosch FX, Lorincz A, Muñoz N, Meijer CJLM, Shah KV. The causal relation between human papillomavirus and cervical cancer, Journal of Clinical Pathology. 2002; 55(4): 244–265. 10.1136/jcp.55.4.244 11919208 PMC1769629

[4] WHO/ICO HPV information centre on HPV and cervical cancer (HPV information centre). Human papillomavirus and related cancers in Nigeria. Summary report 2010. Available: www.who.int/hpvcentre.

[5] Woodman CBJ, Stuart I, Collins SI, Young LS. The natural history of cervical HPV infection: unresolved issues. Nature Reviews Cancer. 2007; 7: 11–22. 10.1038/nrc2050 17186016

[6] Fernandes J, Carvalho M, de Fernandes T, Araújo J, Azevedo P, Azevedo J, et al Prevalence of Human Papillomavirus Type 58 in Women With or Without Cervical Lesions in Northeast Brazil. Annals of Medical and Health Sciences Research. 2013; 3(4): 504–510. 10.4103/2141-9248.122060 24379999 PMC3868114

[7] HPV Center http://www.hpvcenter.se/html/refclones.html. Accessed 29 Dec 2017. Last modified by: Davit Bzhalava 2016-05-24 email: davit.bzhalava@ki.se

[8] de Villiers EM, Fauquet C, Broker TR, Bernard HU, zur Hausen H. Classification of papillomaviruses. Virology. 2004; 324: 17–27. 10.1016/j.virol.2004.03.033 15183049

[9] Lee K, Magalhaes I, Clavel C, Briolat J, Birembaut P, Tommasino M, et al Human papillomavirus 16 E6, L1, L2 and E2 gene variants in cervical lesion progression. Virus Res. 2008; 131: 106–110. 10.1016/j.virusres.2007.08.003 17869365

[10] Villa LL, Sichero L, Rahal P, Caballero O, Ferenczy A, Rohan T, et al Molecular variants of human papillomavirus types 16 and 18 preferentially associated with cervical neoplasia. J Gen Virol. 2000; 81: 2959–2968. 10.1099/0022-1317-81-12-2959 11086127

[11] Xi LF, Koutsky LA, Hildesheim A, Galloway DA, Wheeler CM, Winer RL, et al Risk for high-grade cervical intraepithelial neoplasia associated with variants of human papillomavirus types 16 and 18. Cancer Epidemiol. Biomarkers Prev. 2007; 16: 4–10 10.1158/1055-9965.EPI-06-0670 17220325

[12] Zuna RE, Moore WE, Shanesmith RP, Dunn ST, Wang SS, Schiffman M., et al Association of HPV16 E6 variants with diagnostic severity in cervical cytology samples of 354 women in a US population. Int. J. Cancer. 2009; 125: 2609–2613 10.1002/ijc.24706 19569178 PMC2757470

[13] Muñoz N, Bosch FX, de Sanjosé S, Herrero R, Castellsagué X, Shah KV, et al Epidemiological classification of human papillomavirus types associated with cervical cancer. N. Engl.J.Med. 2003; 348: 518–27. 10.1056/NEJMoa021641 12571259

[14] Clifford G, Franceschi S, Diaz M, Munoz N, Villa LL. Chapter 3: HPV type-distribution in women with and without cervical neoplastic diseases. Vaccine. 2006; 24 (Suppl 3): S3/26–34. 10.1016/j.vaccine.2006.05.026 16950015

[15] Odetola TD, Ekpo K. Community Medicine & Health Education Nigerian Women’s Perceptions about Human Papillomavirus Immunisations. J Community Med Health Educ. 2012; 2(11): 1–5.

[16] Food and Drug Administration (FDA) 2014. Approval letter—GARDASIL 9. Silver Spring, MD: US Department of Health and Human Services, Food and Drug Administration; 12 10, 2014 Available at http://www.fda.gov/BiologicsBloodVaccines/Vaccines/Approved Products/ucm426520.htm

[17] Charan J, Biswas T. How to calculate sample size for different study designs in medical research. Indian J Psychol Med. 2013; 35(2): 121–126. 10.4103/0253-7176.116232 24049221 PMC3775042

[18] Thomas JO, Herrero R, Omigbodun AA, Ojemakinde K, Ajayi IO, Fawole A, et al Prevalence of papillomavirus infection in women in Ibadan, Nigeria: a population-based study. British Journal of Cancer. 2004; 90: 638–645 10.1038/sj.bjc.6601515 14760378 PMC2409602

[19] Sotlar K, Diemer D, Dethleffs A, Hack Y, Stubner A, Vollmer N, et al Detection and typing of human papillomavirus by E6 nested multiplex PCR. Journal of Clinical Microbiology. 2004; 42: 3176–3184. 10.1128/JCM.42.7.3176-3184.2004 15243079 PMC446280

[20] Tamura K, Stecher G, Peterson D, Filipski A, Kumar S. MEGA6: Molecular Evolutionary Genetics Analysis version 6.0. Molecular Biology and Evolution. 2013; 30: 2725–2729. 10.1093/molbev/mst197 24132122 PMC3840312

[21] Tamura K, Nei M. Estimation of the number of nucleotide substitutions in the control region of mitochondrial DNA in Humans and chimpanzees. Molecular Biology and Evolution. 1993; 10: 512–526. 10.1093/oxfordjournals.molbev.a040023 8336541

[22] Nejo YT, Olaleye DO, Odaibo GN. Prevalence and Risk Factors for Genital Human Papillomavirus Infections Among Women in Southwest Nigeria. Archives of basic and applied medicine. 2018; 6(1): 105–112. 29905313 PMC5997288

[23] Schnatz PF, Markelova NV, Holmes D, Mandavilli SR, O’Sullivan DM. The prevalence of cervical HPV and cytological abnormalities in association with reproductive factors of rural Nigerian women. Journal of Womens Health (Larchmt). 2008; 17: 279–285. 10.1089/jwh.2006.0295 18321179

[24] Akarolo-Anthony SN, Al-Mujtaba M, Famooto AO, Dareng EO, Olaniyan OB, Offiong R, et al HIV associated high-risk HPV infection among Nigerian women. BMC Infectious Diseases. 2013; 13: 521 10.1186/1471-2334-13-521 24192311 PMC3826514

[25] Dareng EO, Ma B, Famooto AO, Akarolo-Anthony SN, Offiong RA, Olaniyan O, et al Prevalent high-risk HPV infection and vaginal microbiota in Nigerian women. Epidemiology and Infection. 2016; 144(1): 123–137. 10.1017/S0950268815000965 26062721 PMC4659743

[26] Fadahunsi OO, Omoniyi-Esan GO, Banjo AA, Esimai OA, Osiagwu D, Clement F, et al Prevalence of High Risk oncogenic HPV types in cervical smears of women attending well women clinic in Ile-Ife. Gynaecology Obstetrics. 2013; 3(6): 1000185.

[27] Nweke IG, Banjo AAF, Abdulkareem FB, Nwadike VU. Prevalence of Human Papilloma virus DNA in HIV positive women in Lagos University Teaching Hospital (LUTH) Lagos, Nigeria. British Microbiology Research Journal. 2013; 3(3): 400–413.

[28] Adegbesan-Omilabu MA, Okunade KS, Omilabu SA. Oncogenic human papillomavirus infection among women attending the cytology clinic of a tertiary hospital in Lagos, South-West Nigeria. International Journal of Research in Medical Sciences. 2014; 2(2): 625–630.

[29] Ezechi OC, Ostergren PO, Nwaokorie FO, Ujah IAO, Odberg PK. The burden, distribution and risk factors for cervical oncogenic Human papillomavirus infection in HIV positive Nigerian women. Virology Journal. 2014; 11:15 10.1186/1743-422X-11-15 24433568 PMC3896716

[30] Piras F, Piga M, De Montis A, Zannou AR, Minerba L, Perra MT, et al Prevalence of human papillomavirus infection in women in Benin, West Africa. Virology Journal. 2011; 8: 514 10.1186/1743-422X-8-514 22074103 PMC3231975

[31] Kuhn L, Denny L, Pollack A, Lorincz A, Richart RM, Wright TC. Human papillomavirus DNA testing for cervical cancer screening in low-resource settings. Journal of National Cancer Institute. 2000; 92: 818–825. 10.1093/jnci/92.10.818 10814677

[32] Zohoncon TM, Bisseye C, Djigma FW, Yonli AT, Compaore TR, Sagna T, et al Prevalence of HPV High-Risk Genotypes in Three Cohorts of Women in Ouagadougou (Burkina Faso). Mediterranean Journal of Hematology and Infectious Diseases. 2013. 10.4084/MJHID.2013.059 PMC3787662 24106609

[33] Traore IMA, Zohoncon TM, Dembele A, Djigma FW, Obiri-Yeboah D, Traore G, et al Molecular Characterization of High-Risk Human Papillomavirus in Women in Bobo-Dioulasso, Burkina Faso. BioMed Research International. 2016; 7092583 10.1155/2016/7092583 27525275 PMC4971308

[34] de Sanjosé S, Diaz M, Castellsagué X, Clifford G, Bruni L, Muñoz N, et al Worldwide prevalence and genotype distribution of cervical human papillomavirus DNA in women with normal cytology: a meta-analysis. Lancet Infectious Diseases. 2007; 7(7): 453 59. 10.1016/S1473-3099(07)70158-5 17597569

[35] Bruni L, Diaz M, Castellsagué X, Ferrer E, Bosch FX, de Sanjosé S. Cervical Human Papillomavirus Prevalence in 5 Continents: Meta‐Analysis of 1 Million Women with Normal Cytological Findings. The Journal of Infectious Diseases. 2010; 202(12): 1789–1799. 10.1086/657321 21067372

[36] de Sanjose S, Quint WG, Alemany L, Geraets DT, Klaustermeier JE, Lloveras B, et al Human papillomavirus genotype attribution in invasive cervical cancer: a retrospective cross-sectional worldwide study. Lancet Oncology. 2010; 11:1048–1056. 10.1016/S1470-2045(10)70230-8 20952254

[37] Li N, Franceschi S, Howell-Jones R, Snijders PJ, Clifford GM. Human papillomavirus type distribution in 30,848 invasive cervical cancers worldwide: variation by geographical region, histological type and year of publication. International Journal of Cancer. 2011; 128: 927–935. 10.1002/ijc.25396 20473886

[38] Denny L, Adewole I, Anorlu R, Dreyer G, Moodley M, Smith T, et al Human papillomavirus prevalence and type distribution in invasive cervical cancer in sub-Saharan Africa. International Journal of Cancer. 2014; 134: 1389–1398. 10.1002/ijc.28425 23929250

[39] Ali-Risasi C, Verdonck K, Padalko E, Vanden BD, Praet M. Prevalence and risk factors for cancer of the uterine cervix among women living in Kinshasa, the Democratic Republic of the Congo: a cross-sectional study. Infectious Agents and Cancer. 2015; 10: 20 10.1186/s13027-015-0015-z 26180542 PMC4502934

[40] Kennedy NT, Ikechukwu D, Goddy B. Risk factors and distribution of oncogenic strains of human papilloma virus in women presenting for cervical cancer screening in Port Harcourt, Nigeria. The Pan African medical journal. 2016; 23: 85 10.11604/pamj.2016.23.85.8510 27222684 PMC4867190

[41] Gravitt PE, Kamath AM, Gaffikin L, Chirenje ZM, Womack S, Shah KV. Human papillomavirus genotype prevalence in high-grade squamous intraepithelial lesions and colposcopically normal women from Zimbabwe. International Journal of Cancer. 2002; 100: 729–732. 10.1002/ijc.10538 12209615

[42] Yamada T, Manos MM, Peto J, Greer CE, Munoz N, Bosch FX, et al Human papillomavirus type 16 dsequence variation in cervical cancers: a worldwide perspective. J Virol Methods. 1997; 71: 2463–2472. 10.1128/jvi.71.3.2463-2472.1997 PMC191357 9032384

[43] Cornet I, Gheit T, Iannacone MR, Vignat J, Sylla BS, Del Mistro A, et al HPV16 genetic variation and the development of cervical cancer worldwide. Br J Cancer. 2013; 108: 240–244. 10.1038/bjc.2012.508 23169278 PMC3553516

[44] Cornet I, Gheit T, Franceschi S, Vignat J, Burk RD, Sylla BS, et al Human Papillomavirus Type 16 Genetic Variants: Phylogeny and Classification Based on E6 and LCR. J Virol. 2012; 86: 6855–6861. 10.1128/JVI.00483-12 22491459 PMC3393538

[45] Boumba LMA, Assoumou SZ, Hilali L, Mambou JV, Moukassa D, Ennaji MM. Genetic variability in E6 and E7 oncogenes of human papillomavirus Type 16 from Congolese cervical cancer isolates. Infectious Agents and Cancer. 2015; 10(1):15. 25991921 10.1186/s13027-015-0010-4 PMC4437748

[46] Schlecht NF, Burk RD, Palefsky JM, Minkoff H, Xue X, Massad LS, et al Variants of human papillomaviruses 16 and 18 and their natural history in human immunodeficiency virus-positive women. Journal of General Virology. 2005; 86: 2709–2720. 10.1099/vir.0.81060-0 16186224

[47] Smith B, Chen Z, Reimers L, van Doorslaer K, Schiffman M, DeSalle R, et al Sequence imputation of HPV16 genomes for genetic association studies. PLoS ONE. 2011; 6: 10.1371/journal.pone.0021375 21731721 PMC3121793

[48] Burk RD, Harari A, Chen Z. Human papillomavirus genome variants. Virology. 2013; 445: 232–243. 10.1016/j.virol.2013.07.018 23998342 PMC3979972

[49] Pérez S, Cid A, Iñarrea A, Pato M, Lamas MJ, Couso B, et al Prevalence of HPV 16 and HPV 18 Lineages in Galicia, Spain. PLoS ONE. 2014; 9(8): e104678 10.1371/journal.pone.0104678 25111834 PMC4128731

[50] Tu JJ, Kuhn L, Denny L, Beattie KJ, Lorincz A, Wright TC. Molecular variants of human papillomavirus type 16 and risk for cervical neoplasia in South Africa. Int J Gynecol Cancer. 2006; 16: 736–742. 10.1111/j.1525-1438.2006.00401.x 16681754

[51] Qmichou Z, Ennaji MM, Amrani M, Fahime EM, Meloul M, Meftah EL, et al Molecular Characterization of HPV16 E6 and E7 Variants among Women with Cervical Cancer in Moroco. Britsh Microbiol Res J. 2013; 3: 692–705

[52] Bernard HU, Calleja-Macias IE, Dunn ST. Genome variation of human papillomavirus types: phylogenetic and medical implications. Int J Cancer. 2006; 118: 1071–1076. 10.1002/ijc.21655 16331617

[53] Sichero L, Ferreira S, Trottier H, Duarte-Franco E, Ferenczy A, Franco EL, et al High grade cervical lesions are caused preferentially by non-European variants of HPVs 16 and 18. Int J Cancer. 2007; 120: 1763–1768. 10.1002/ijc.22481 17230525

[54] Freitas LB, Chen Z, Muqui EF, Boldrini NAT, Miranda AE, Spano LC, et al Human Papillomavirus 16 Non-European variants are preferentially associated with high-grade cervical lesions. PLoS One. 2014; 9(7): e100746 10.1371/journal.pone.0100746 24983739 PMC4077691

[55] Crook T, Tidy JA, Vousden KH. Degradation of p53 can be targeted by HPV E6 sequences distinct from those required for p53 binding and trans-activation. Cell. 1991; 67: 547–556. 10.1016/0092-8674(91)90529-8 1657399

[56] Stöppler MC, Ching K, Stöppler H, Clancy K, Schlegel R, Icenogle J. Natural variants of the human papillomavirus type 16 E6 protein differ in their abilities to alter keratinocyte differentiation and to induce p53 degradation. J Virol. 1996; 70: 6987–6993. 8794343 10.1128/jvi.70.10.6987-6993.1996 PMC190749

[57] Ellis JRM, Etherington I, Galloway D, Luesley D, Young LS. Antibody responses to HPV16 virus-like particles in women with cervical intraepithelial neoplasia infected with a variant HPV16. The Lancet. 1997; 349: 1069–1070. 10.1016/s0140-6736(05)62292-1 9107250

[58] Chow VT, Loh E, Yeo WM, Tan SY, Chan R. Identification of multiple genital HPV types and sequence variants by consensus and nested type-specific PCR coupled with cycle sequencing. Pathology. 2000; 32: 204–208. 10968397

[59] Shen M, Ding X, Li T, Chen G, Zhou X. Sequence Variation Analysis of HPV-18 Isolates in Southwest China. PLoS ONE. 2013; 8: 10.1371/journal.pone.0056614 23451059 PMC3581518

[60] Arroyo SL, Basaras M, Arrese E, Hernáez S, Andía D, Esteban V, et al Human Papillomavirus (HPV) genotype 18 variants in patients with clinical manifestations of HPV related infections in Bilbao, Spain. Virology Journal. 2012; 9: 258 10.1186/1743-422X-9-258 23121839 PMC3495774

[61] Kovelman R, Bilter GK, Roman A, Brown DR, Barbosa MS. Human papillomavirus type 6: classification of clinical isolates and functional analysis of E2 proteins. Journal of General Virology. 1999; 80: 2445–2451. 10.1099/0022-1317-80-9-2445 10501500

[62] Kocjan BJ, Poljak M, Cimerman M, Gale N, Potočnik M, Bogovac Ž, et al Prevaccination genomic diversity of human papillomavirus genotype 6 (HPV 6). *Virology* 2009; 391: 274–283. 10.1016/j.virol.2009.06.030 19596128

[63] Danielewski JA, Garland SM, McCloskey J, Hillman RJ, Tabrizi SN. Human Papillomavirus Type 6 and 11 Genetic Variants Found in 71 Oral and Anogenital Epithelial Samples from Australia. PLoS ONE. 2013; 8(5). 10.1371/journal.pone.0063892 PMC3656832 23691108

[64] Gagnon S, Hankins C, Tremblay C, Pourreaux K, Forest P, Rouah F, et al Polymorphism of human papillomavirus type 31 isolates infecting the genital tract of HIV-seropositive and HIV-seronegative women at risk for HIV infection. Journal of Medical Virology. 2005; 75: 213–221. 10.1002/jmv.20259 15602735

[65] Calleja-Macias IE, Kalantari M, Allan B, Williamson AL, Chung LP, Collins RJ, et al Papillomavirus subtypes are natural and old taxa: phylogeny of human papillomavirus types 44 and 55 and 68a and -b. Journal of virology. 2005; 79: 6565–6569. 10.1128/JVI.79.10.6565-6569.2005 15858044 PMC1091730

[66] Chen Z, Schiffman M, Herrero R, DeSalle R, Anastos K, Segondy M, et al Evolution and taxonomic classification of human papillomavirus 16 (HPV16)-related variant genomes: HPV31, HPV33, HPV35, HPV52, HPV58 and HPV67. PLoS ONE. 2011; 6: 10.1371/journal.pone.0020183 21673791 PMC3103539

[67] Maver PJ, Kocjan BJ, Seme K, Poljak M. Genomic diversity of low-risk human papillomavirus genotypes HPV 40, HPV 42, HPV 43, and HPV 44. Journal of Medical Virology. 2014; 86(2), 272–282. 10.1002/jmv.23822 24155245

[68] de Villiers EM. Heterogeneity of the human papillomavirus group. Journal of Virology. 1989; 63: 4898–4903.4. 2552162 10.1128/jvi.63.11.4898-4903.1989 PMC251129

[69] Prado JC, Calleja-Macias IE, Bernard HU, Kalantari M, Macay SA, Allan B, et al Worldwide genomic diversity of the human papillomaviruses-53, 56, and 66, a group of high-risk HPVs unrelated to HPV-16 and HPV-18. Virology. 2005; 340: 95–104. 10.1016/j.virol.2005.06.024 16039686

[70] Chang YJ, Chen HC, Lee BH, You SL, Lin CY, Pan MH, et al Unique variants of human papillomavirus genotypes 52 and 58 and risk of cervical neoplasia. International Journal of Cancer. 2011; 129(4): 965–973. 10.1002/ijc.25724 20949622

[71] Choi YD, Han CW, Chung WJ et al, “Analysis of HPV-other samples by performing HPV DNA sequencing,” Korean Journal of Pathology. 2009; 43(3): 250–253.

[72] Ngai Na Chloe Co, Lai-On C, Joseph KFC, Joseph WOT, Enders KON. HPV Prevalence and Detection of Rare HPV Genotypes in Hong Kong Women from Southern China with Cytological Abnormalities. ISRN Virology. 2013 10.5402/2013/312706.

[73] Molden T, Feiring B, Ambur OH, et al Human papillomavirus prevalence and type distribution in urine samples from Norwegian women aged 17 and 21 years: A nationwide cross-sectional study of three non-vaccinated birth cohorts. Papillomavirus Res. 2016;2:153–158. 10.1016/j.pvr.2016.05.002 29074174 PMC5886875

[74] Longuet M, Cassonnet P, Orth G. A novel genital human papillomavirus (HPV), HPV type 74, found in immunosuppressed patients. J Clin Microbiol. 1996; 34(7): 1859–1862. 8784613 10.1128/jcm.34.7.1859-1862.1996 PMC229138

[75] Terai M, and Burk RD. Characterization of a novel genital human papillomavirus byoverlapping PCR: candHPV86 identified in cervicovaginal cells of a woman with cervical neoplasia. Journal of General Virology. 2001; 82: 2035–2040. 10.1099/0022-1317-82-9-2035 11514712

